# Study on the Rheological Behaviors, Thixotropy, and Printing Characteristics of Screen Printing Slurry for Nd-Fe-B

**DOI:** 10.3390/ma17184626

**Published:** 2024-09-20

**Authors:** Xiaojun Sun, Xiao Lin, Yang Luo, Dunbo Yu, Wenlong Yan, Hongbin Zhang, Zilong Wang, Chaofan Zhang, Jiyuan Guo, Wendi Zhang, Weiguo Gao, Shan Huang

**Affiliations:** 1 National Engineering Research Center for Rare Earth, Grirem Hi-Tech Co., Langfang 065201, China; linxiao@grirem.com (X.L.); yudunbo@griam.cn (D.Y.); yanwl@grirem.com (W.Y.); zhanghb@grirem.com (H.Z.); wangzl@grirem.com (Z.W.); zhangwd1615@163.com (W.Z.); gaowg20212021@163.com (W.G.); huangshansix@163.com (S.H.); 2 Grirem (Rongcheng) Co., Ltd., Weihai 264306, China; zhangcf@grirem.com (C.Z.); guojy@grirem.com (J.G.)

**Keywords:** Nd-Fe-B, screen printing, slurry, rheological behavior, thixotropy

## Abstract

The rheological behavior and printing characteristics of the screen-printing slurry for Nd-Fe-B grain boundary diffusion are key factors that determine the quality of printing and magnetic performance. However, few studies have focused on the organic medium, a crucial material for slurry. In this paper, the rheology, thixotropy, and thermal decomposition behavior of the organic vehicle in Nd-Fe-B screen printing slurry were studied. The results show that the organic vehicle formed by terpineol and polyvinyl butyral (PVB) exhibits typical non-Newtonian fluid characteristics, with excellent rheology and thixotropy, ensuring that the slurry prepared from it has excellent static stability and printing consistency. Additionally, the carbon residue of the organic vehicle formed by terpineol and PVB is less than 0.1% at 900 °C, avoiding excessive carbon entering the magnet during the diffusion process. Moreover, studying the rheology and thixotropy of the organic vehicle through a rheometer can quickly screen the slurry system. This work provides valuable guidance for designing an organic vehicle for screen-printing slurry for Nd-Fe-B grain boundary diffusion in future research.

## 1. Introduction

Nakamura et al. [1] proposed a grain boundary diffusion process (GBDP), which typically involves covering the surface of the original Nd-Fe-B magnet with a diffusion source, followed by heat treatment, to allow the diffusion source to penetrate into the magnet along the liquid Nd-rich phase. GBDP can significantly enhance the coercivity (*H*_cj_) of Nd-Fe-B permanent magnetic materials without significantly reducing the remanence (*B*_r_) and maximum magnetic energy product ((*BH*)_max_). Recently, a large number of studies have been conducted on the GBDP of Nd-Fe-B magnets, leading to the development of multiple coating technologies, each with its own advantages and disadvantages [2,3,4,5,6]. Vacuum evaporation produces diffusion sources with high purity, but the adhesion between the diffusion source and the substrate is poor, making it prone to falling off [7]. Electroplating could exert control over the thickness of the diffusion source on the magnet surface, but the deposition efficiency decreases sharply over time [8]. Dipping offers the advantage of simplicity in the process, suitable for mass production, but it is difficult to ensure uniformity in the coating and can result in a waste of rare earth elements [9]. Magnetron sputtering deposits a diffusion source that adheres well to the substrate and has high purity, but the target material (heavy rare earth elements such as Tb or Dy) utilization ratio is low and the equipment is very expensive [10].

To address the shortcomings of existing grain boundary diffusion methods, screen printing technology was applied in the Nd-Fe-B industry [11,12]. Screen printing involves using a screen stencil and slurry to print the slurry onto the substrate through the holes of the stencil by scraping. Screen printing integrates the advantages of sputtering and dipping methods, achieving the effects of magnetron sputtering and dipping with low cost and high production efficiency, and has become one of the mainstream processes in the industry. The slurry used for Nd-Fe-B screen printing mainly consists of rare earth powders (heavy rare earth metals, heavy rare earth compounds, and heavy rare earth alloys), organic solvents, binders, and additives [13,14,15,16]. Organic solvents, binders, and additives together form the organic vehicles. The primary role of the organic vehicles is to wet the diffusion source powder, turning it into a flowable slurry, which plays a decisive role in the rheology and printing performance of the slurry [17,18,19]. Among organic vehicles, the organic solvent primarily provides polar groups that can dissolve the binders, and the binders mainly increase the viscosity of the organic vehicles and slurry [20,21,22]. As a key ingredient affecting the printing of the slurry, firstly, the viscosity of the organic vehicle must be appropriate. Too high a viscosity makes it difficult to pass through the screen during printing, while too low a viscosity causes it to stick to the screen. Secondly, the organic vehicle must have excellent rheological properties to ensure printing stability. Thirdly, the organic vehicle should not easily evaporate under storage conditions to prevent solvent exudation, and it should evaporate evenly at high temperatures to prevent the film layer peeling that may occur during concentrated evaporation, leading to a decrease in diffusion effect. Finally, the organic vehicle needs to have excellent wettability for the diffusion source powder, allowing solid particles to be evenly dispersed without agglomeration and sedimentation [23,24].

Current research on the organic vehicle components of Nd-Fe-B screen printing slurry is limited, with a focus on printing consistency rather than the rheological behavior and other characteristics of the slurry itself. This has resulted in slow progress in related research and high trial-and-error costs. Therefore, this paper delves into the impact of organic components on the rheological behavior and printing properties of the slurry, introducing a new method to accelerate the selection of organic vehicles using rheometers and other instruments, providing new ideas and methods for subsequent slurry research.

## 2. Material and Methods

**Figure 1:**
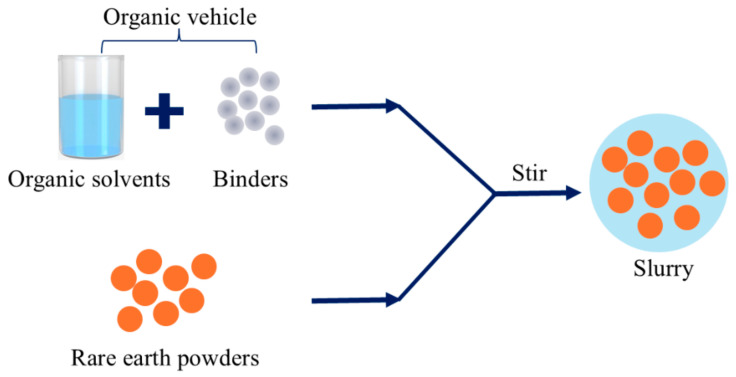
Schematic diagram of the preparation process.

We weighed a certain amount of organic solvent according to the proportion and added the binder while stirring. After all the binders were added, we continued to stir at 1200 r/min for 15 min to ensure that all the binders were dissolved in the organic solvent. Subsequently, under a nitrogen atmosphere, we added the diffusion source powder to the prepared organic vehicle and stirred at 1500 r/min for 30 min to ensure the uniformity of the slurry. The schematic diagram of the preparation process is shown in Figure 1.

The static stability of the slurry was studied using an Imhoff cone settling cup. Steady viscosity curves were recorded by controlling the shear rate from 0.1 to 1000 s^−1^ at 25 °C by using a rheometer (MARS60, Haake, Karlsruhe, Germany). To study the structural destruction and reconstruction of the organic vehicle during screen printing, the three-interval thixotropy shear test was used to simulate the behavior during printing. In this test, the shear rate increased from 0.1 to 500 s^−1^ and then reversed back to 0.1 s^−1^. The organic vehicle was heated from 25 to 400 and 900 °C at a rate of 20 °C/min in a nitrogen atmosphere by using a thermogravimetric analyzer, and the residual carbon in the residue was analyzed by an organic element analyzer (Thermo Scientific Flash 2000, Thermo Scientific, Waltham, MA, USA). To ensure the accuracy of the experimental results, all the above measurements were repeated three times.

## 3. Results and Discussion

### 3.1. The Effect of Composition on the Rheological Behavior of Organic Vehicles

#### 3.1.1. Influence of Organic Solvent on the Rheological Properties

**Figure 2:**
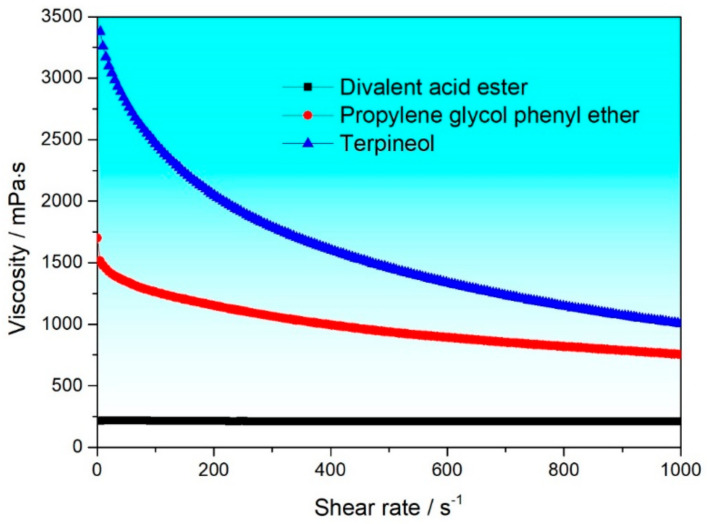
Shear rate-viscosity curves of different organic solvents.

The slurry used for Nd-Fe-B screen printing mainly consists of rare earth powder, organic solvents, binders, and additives, among which the organic solvent directly determines the stability, volatility, and other properties of the slurry. Therefore, this paper first studies the impact of organic solvents on the rheological properties of the slurry. According to solvents used commonly in industrial production, polyvinyl butyral (PVB) with a mass fraction of 6% was added to terpineol, propylene glycol phenyl ether (PPH), and divalent acid ester (DBE), respectively, and then allowed to stand after high-speed dispersion. Figure 2 shows the viscosity curves of the organic vehicles prepared with three different organic solvents. It can be seen that the viscosities of the two types of organic vehicle prepared with terpineol and PPH both decreased with the increase in shear rate. The results show that the terpineol and PPH systems possess a shear thinning property, so both types of organic vehicle are non-Newtonian fluids, meeting the requirements for use in slurries [25,26,27]. This is because this characteristic allows the viscosity of the slurry to decrease with an increase in shear rate when subjected to shear action, thereby making it easier to pass through the fine pores of the screen printing and achieve a uniform coating. Moreover, the internal microstructure of non-Newtonian fluids changes when subjected to shear, and this change helps the slurry adapt better to complex surface shapes during the printing process, improving the quality and efficiency of printing. For the DBE system, the viscosity does not change significantly with the increase in shear rate, indicating that DBE systems belong to Newtonian fluids, which is not suitable for preparing slurries.

#### 3.1.2. Effect of Binder Type on the Thixotropy

**Figure 3:**
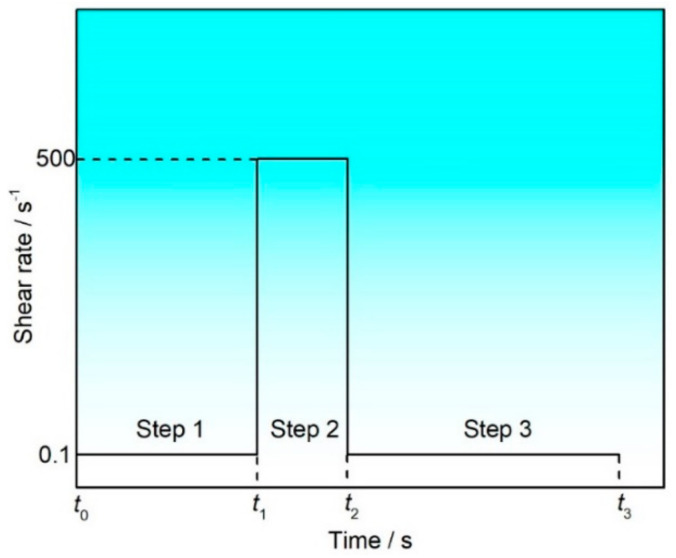
Schematic diagram of three-stage shear test.

**Figure 4:**
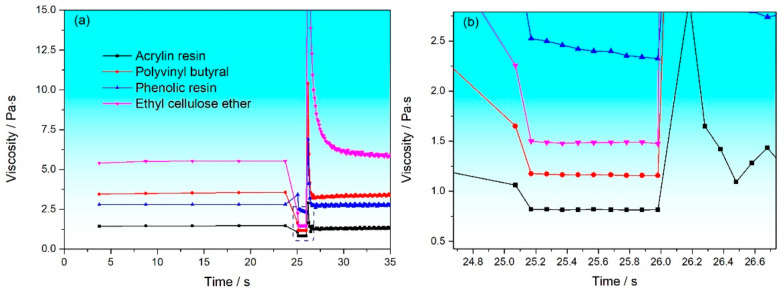
(**a**) Thixotropic curves of terpineol with different resins added, (**b**) localized enlarged view under high-speed shear state.

To study the structural damage and reconstruction of organic vehicles in the screen printing process, the three-interval thixotropy shear test was used to simulate the entire process, as shown in Figure 3. The entire printing process is divided into three steps: the first step simulates the slurry storage before printing with a shear rate of 0.1 s^−1^, during which the slurry is at rest and almost unaffected by any shear force. The second step simulates the behavior of the slurry during the printing process. The slurry is compressed and forced to deform, and the internal spatial network structure is destroyed by external forces, thereby reducing the viscosity of the slurry and starting to flow, with the shear rate set at 500 s^−1^ for this segment. The third step simulates the structural recovery process of the slurry after printing, with the shear rate returned to 0.1 s^−1^. According to the results of the shear rate–viscosity curve, terpineol and PPH were selected as organic solvents and different resins were added to study their three-interval thixotropy shear behavior. Figure 4a shows the structural damage and reconstruction of organic vehicles prepared by terpineol with different resins during the printing process simulated by using a three-interval thixotropy shear test, and Figure 4b is a local enlargement of Figure 4a under high shear rates (the dashed box area in Figure 4a). The results indicate that the four types of organic vehicle are horizontal in the initial stage, showing that the organic vehicles are relatively stable in the static state. Under high-speed shear conditions, although the viscosities of the four types of organic vehicle are around 2 Pa·s, the viscosity of the organic vehicles prepared by terpineol and phenolic resin (PF) shows a continuous downward trend, which is not conducive to the stability of the printing process, while the viscosities of the other three types of organic vehicle are relatively stable under high-speed shear conditions.

**Figure 5:**
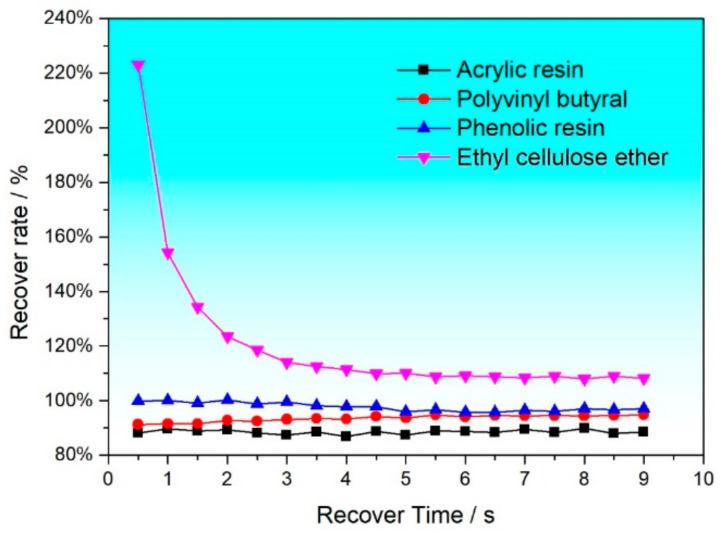
Recovery ratio of organic vehicles within the specified time in the three-stage shear test.

The third step of the three-stage variable shear test simulates the structural recovery process of the slurry after printing, and the structural recovery ratio of the organic vehicle is calculated through the formula $recovery\;ratio\;\left(\%\right)\;=\;\frac{viscosity\;after\;high\;shear}{initial\;viscosity}\;\times \;100$, as shown in Figure 5 and Table 1. The results indicate that the organic vehicles prepared with PVB always exhibit the best recovery performance within the specified time. Within 1 s after the removal of external force, the recovery ratio of the organic vehicles prepared with PVB is 91.6%, and it reaches 94.8% at 9 s. This characteristic not only ensures the stability of production batches but also guarantees that the slurry can quickly recover from a low viscosity state to a high viscosity state after passing through the mesh, thereby maintaining a stable printing pattern. Although these data are lower than the recovery ratio of the organic vehicles prepared with PF (100% recovery ratio at 1 s and 97.1% at 9 s), the organic vehicles prepared with PF show a decreasing and fluctuating trend in viscosity during the recovery process, indicating that the structure of these organic vehicles is difficult to return to the initial state in a short time after printing, which is not conducive to continuous mass production in industry. Similarly, the organic vehicles with added acrylic resin (AR) and ethyl cellulose ether (EC) are also difficult to return to the initial state in a short time. The above analysis shows that PVB as a binder can combine well with the organic solvent terpineol to form a spatial network structure. The rheological properties of the organic vehicle largely depend on the interaction between the binder and the solvent.

**Figure 6:**
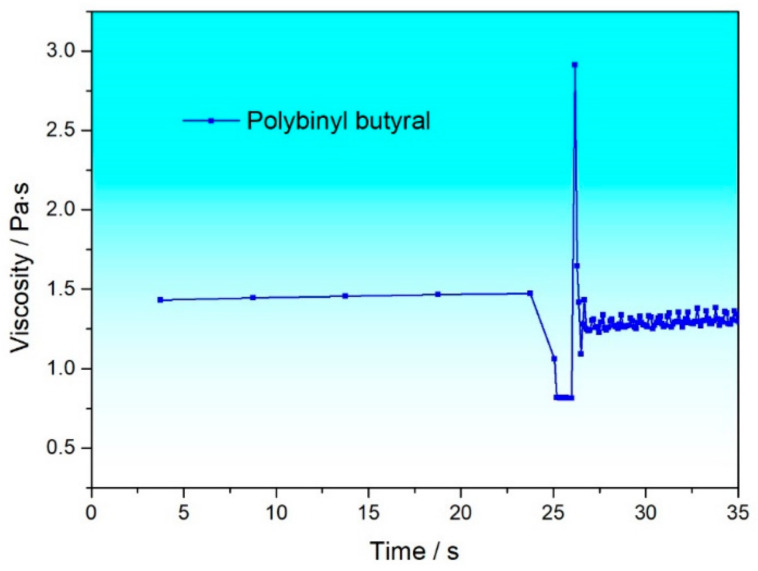
Thixotropic curves of PPH with PVB added.

As for the PPH system with added PVB, although its viscosity curve shows non-Newtonian fluid characteristics, the three-interval thixotropy shear test shows that its viscosity never returns to the initial viscosity during the recovery stage (with only an 89.6% recovery ratio at 9 s, as shown in Figure 6), which will lead to unstable printing. Therefore, PPH is not suitable as a printing organic solvent. Moreover, experimental results show that viscosity testing alone cannot determine whether the organic vehicle is suitable for preparing slurry, and a three-interval thixotropy shear test is also needed.

### 3.2. The Stability and Printing Characteristics of Slurry

**Figure 7:**
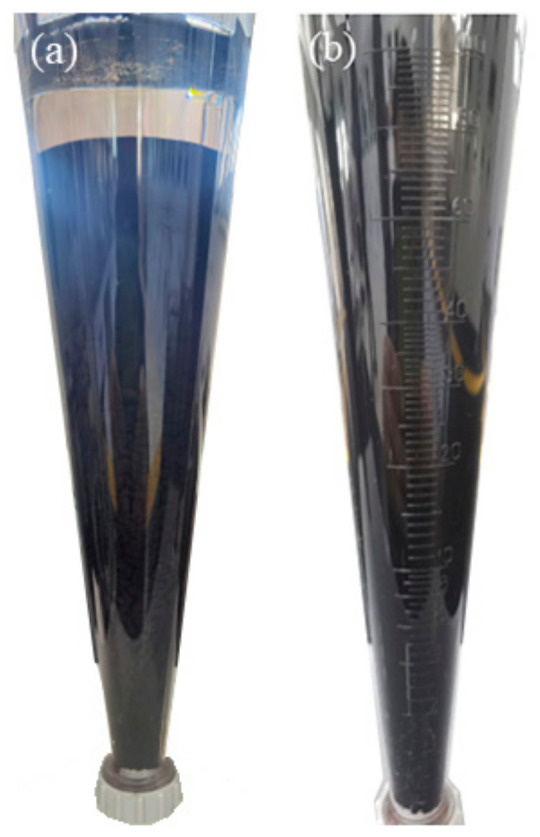
(**a**) PPH and (**b**) terpineol system slurries after 30 days of standing.

**Figure 8:**
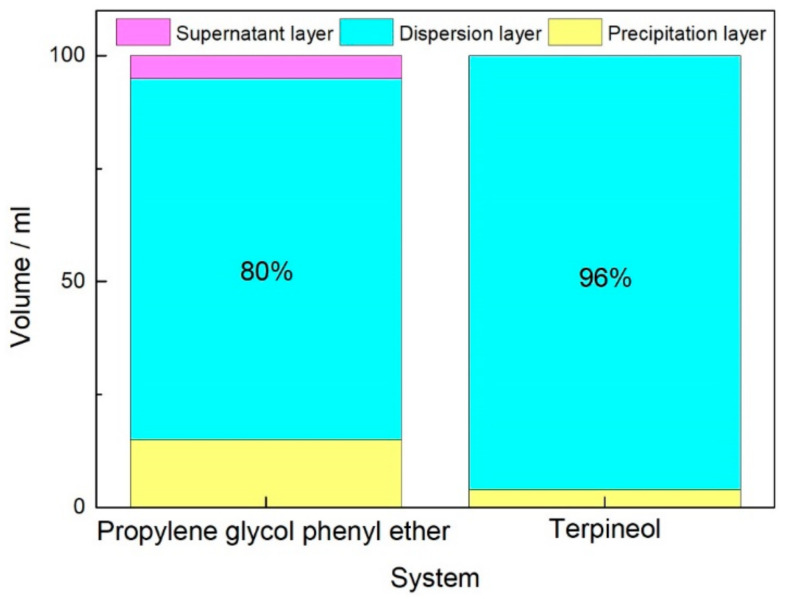
Statistics of supernatant, dispersion, and precipitation layer volume fraction.

Based on the experimental results in Section 3.1, terpineol was selected as the organic solvent and PVB as the binder to further validate the feasibility of determining the quality of slurry through evaluating the rheological and thixotropic properties of the organic vehicle. Terpineol and PVB were prepared in a ratio of 94:6 to make the organic vehicle, which was then mixed with the diffusion source powder at a 2:8 ratio to produce the slurry. The static stability of the slurry was studied using an Imhoff cone settling cup. Figure 7b shows the sedimentation result of the terpineol system slurry after standing for 30 days. Compared to the PPH system (Figure 7a), the suspension performance of the terpineol system was excellent, with no significant precipitation observed after standing for one month. Statistics were conducted on the volume fractions of the supernatant layer, dispersion layer, and precipitation layer, with the results shown in Figure 8. It is evident that the terpineol system slurry showed no significant supernatant after standing for one month, compared to the PPH system, and the volume fraction of the precipitation layer was about 4% (the precipitation layer was mainly composed of the rare earth powders), demonstrating excellent static stability.

In addition to static stability, the stability during the printing process is another important parameter, which is directly related to the consistency of production batches. Table 2 shows the statistical results of the percentage of printing weight to substrate weight with an automatic screen printer. It can be seen that the terpineol system selected based on the rheological properties and thixotropy of the organic vehicle had excellent stability. This further confirms that suitable organic vehicle components can be quickly screened through the study of the rheology and thixotropy of the organic vehicle.

### 3.3. The Thermal Decomposition Behavior of Slurry

Due to the high sensitivity of the Nd-Fe-B matrix to carbon content, if organic residues from coatings decompose into carbon during subsequent diffusion processes and enter the matrix, this will lead to a decline in the magnetic and machining performance of the magnet. By using a thermogravimetric analyzer under a nitrogen atmosphere, the organic vehicle was burned at 400 °C and 900 °C, respectively, and the carbon residue of the organic vehicle was tested through organic element analysis, as shown in Table 3. It can be seen that at the 400 °C degassing stage, the carbon residue after the decomposition of the organic vehicle was only 1.39%, and with a further increase in temperature, the carbon residue at 900 °C was less than 0.01%, indicating that the designed organic vehicle system can be completely thermally decomposed without leaving a large amount of carbon to affect the performance of the magnet. In addition, the grain boundary diffusion effects of screen printing and magnetron sputtering were compared with N48H as the matrix. The coercivity increment of the screen-printed magnet after diffusion reached 10.05 kOe, consistent with the coercivity increment of 10.11 kOe for the magnetron sputtered magnet after diffusion. It can be seen that, under the same condition, the screen-printed diffusion magnet can achieve the same diffusion effect as the magnetron sputtered diffusion magnet.

## 4. Conclusions

In this paper, through rheological and three-stage shear tests, it was found that the organic vehicle formed by terpineol and PVB is suitable for preparing Nd-Fe-B screen printing slurry, and its rheology, thixotropy, and thermal decomposition behavior were studied. The results show that the system exhibits typical non-Newtonian fluid characteristics and has excellent thixotropy, with a recovery ratio of 95% after the external force is removed for 9 s. At the same time, the system has excellent static stability, with a settling ratio of only 4% after 30 days of standing. Moreover, it has excellent printing consistency, which ensures the storage and use of the slurry. The slurry prepared using this system for grain boundary diffusion can achieve the same effect as magnetron sputtering. In addition, the above results prove that studying the rheology and thixotropy of organic vehicles through rheometers can quickly screen slurry systems. Our work provides valuable guidance for designing an organic vehicle for screen-printing slurry for Nd-Fe-B grain boundary diffusion or other areas in future research.

## Figures and Tables

**Table 1:** Recovery ratio of organic vehicles with addition of different resins.

Resin	1 s	3 s	5 s	7 s	9 s
AR	89.7%	87.4%	87.4%	89.4%	88.6%
PVB	91.6%	93.1%	93.6%	94.4%	94.8%
PF	100%	99.4%	95.8%	96.4%	97.1%
EC	154.3%	114%	110%	108.4%	108.2%

**Table 2:** Stability for continuous printing.

Resin	Percentage of Printing Weight to Substrate Weight
Maximum	0.564
Minimum	0.524
Average	0.542

**Table 3:** Carbon residues in organic vehicles at different temperatures.

Temperature	Carbon Residue/%
400 °C	1.391
	1.399
	1.386
900 °C	<0.01
	<0.01
	<0.01

## Data Availability

Data will be made available on request.
